# Addendum: A neural circuit for wind-guided olfactory navigation

**DOI:** 10.1038/s41467-024-46225-8

**Published:** 2024-03-01

**Authors:** Andrew M. M. Matheson, Aaron J. Lanz, Ashley M. Medina, Al M. Licata, Timothy A. Currier, Mubarak H. Syed, Katherine I. Nagel

**Affiliations:** 1 Neuroscience Institute, NYU Medical Center, 435 E 30th St., New York, NY 10016 USA; 2 Center for Neural Science, NYU, New York, NY, 4 Washington Place, New York, NY 10003 USA; 3 Department of Biology, 219 Yale Blvd NE, University of New Mexico, Albuquerque, NM 87131 USA; 4 Present Address: Department of Biological Sciences, Columbia University, 600 Sherman Fairchild Center, New York, NY 10027 USA; 5 Present Address: Department of Neurobiology, Stanford University, 299W. Campus Drive, Stanford, CA 94305 USA

**Keywords:** Navigation, Neural circuits, Olfactory system, Computational neuroscience

Addendum to: *Nature Communications* 10.1038/s41467-022-32247-7, published online 08 August 2022

In our published Article, we identified a Gal4 line (VT062617) that we believed labeled h∆C interneurons of the fan-shaped body. We showed that fan-shaped body interneurons labeled by this line responded to odor in a wind-direction-dependent manner and that both activation and silencing of neurons labeled by this line had effects on fly navigation behavior. Following the publication of the Article, we performed additional experiments to label single neurons within this line, now published in Hamid et al. 2024^1^. These experiments suggest that this line additionally or perhaps predominantly labels a different fan-shaped body interneuron type known as h∆K, which is characterized by a projection to the ellipsoid body. Thus, the sensory responses (Figure 5E–I) and behavioral effects (Figure 6) that we described in the original Article may arise from h∆K rather than h∆C interneurons. In contrast to h∆C, which receives direct projections from the olfactory neuron FB5AB and from wind-sensitive PFNa neurons, as shown in Figure 5B, C of the original Article, h∆K interneurons do not receive direct projections from these two neuron types, although they do receive indirect inputs from them. Thus, the sensory responses shown in Figure 5E–I in line VT062617 may arise through indirect pathways.
