# Supplementary material for: A neural circuit for wind-guided olfactory navigation
Source: Nat Commun. 2022 Aug 8;13:4613. doi: 10.1038/s41467-022-32247-7 (PMC9360402; doi:10.1038/s41467-022-32247-7)
Supplement: Supplementary file 1 — Supplementary Information [file 41467_2022_32247_MOESM1_ESM.pdf]

Supplementary Figure 1

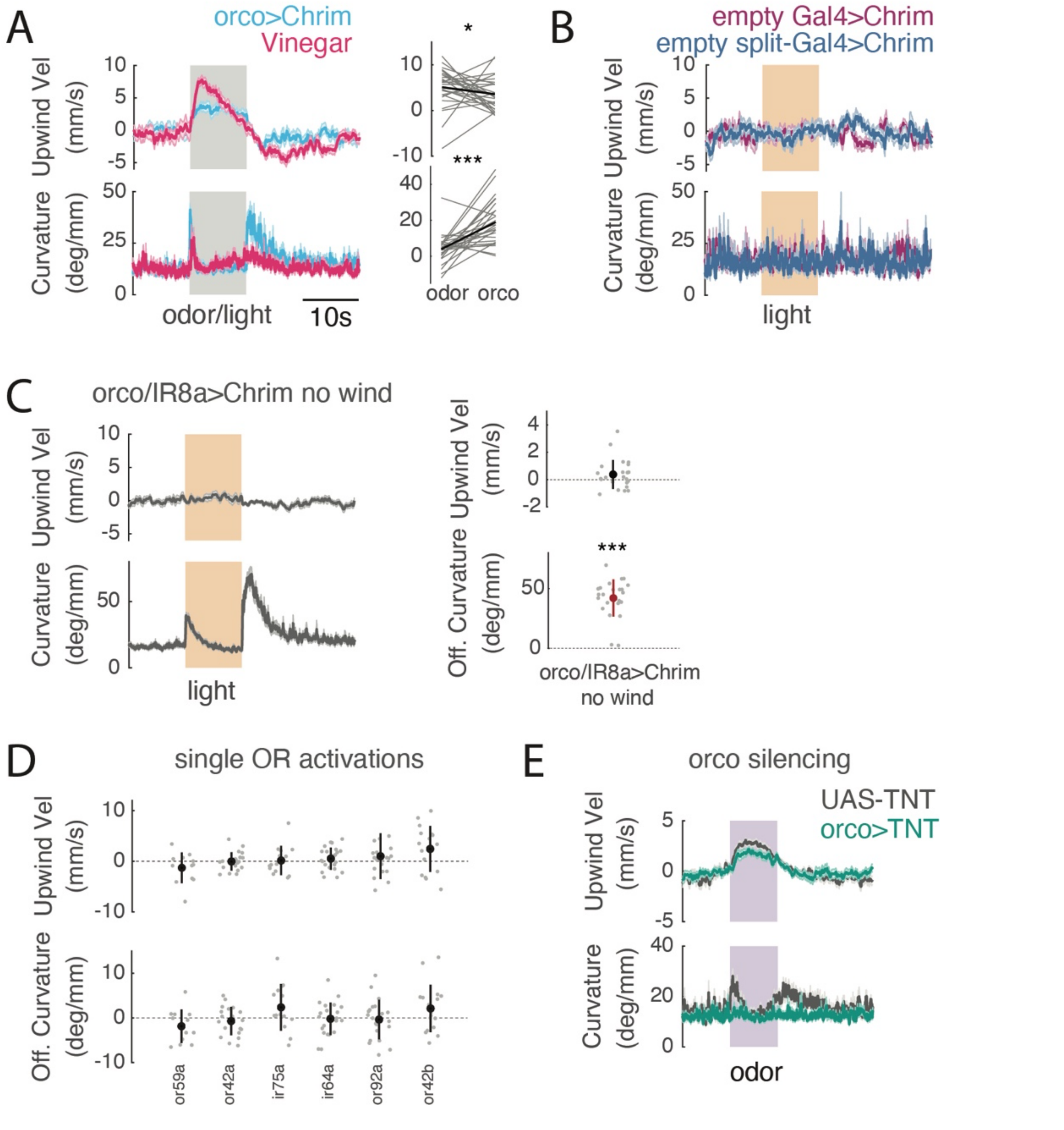

Fig. S1: Additional data on ORN encoding of olfactory navigation behavior

A) Left: Upwind velocity and curvature time courses in the same flies (N = 26 flies) exposed to both 1% vinegar (pink) and optogenetic stimulation of orco+ ORNs (blue) in interleaved trials. Right: Comparison of average upwind velocity (0-5s after stimulus ON) and OFF curvature (0-2s after stimulus OFF) for

each fly. Within each fly, vinegar evoked greater upwind velocity than orco stimulation ($p=0.0319$) but weaker OFF curvature than orco stimulation ($p=1.3216e-04$).

B) Upwind velocity and curvature time courses for control flies: empty-GAL4>Chrimson (purple, N= 19) and empty split-GAL4>Chrimson (blue, N= 14). No significant change in either parameter during stimulation (quantified as in Figure 1D).

C) Right: Upwind velocity and curvature time courses for orco/IR8a>Chrimson flies in the absence of wind (N= 24 flies, see also Alvarez-Salvado et al., 2018 and Suver et al., 2019). Left: quantification as in Fig. 1D. No increase in upwind velocity ($p= 0.0191$), but OFF curvature increases significantly increases after stimulation ($p= 1.8215e-05$).

D) Upwind velocity and OFF curvature (average change from baseline for single flies, mean ±STD overlaid) for each single ORN that responds to vinegar (Jung et al., 2015). No significant increase in upwind velocity (or59a (N=12): p= 0.2334, or42a(N=20): p=0.7089, ir75a (N=14): p=0.9032, ir64a (N=23): p=0.3304, or92a (N=21): p=0.3754, or42b(N=17): p=0.0442) or OFF curvature (or59a: p= 0.1763, or42a: p=0.3317, ir75a: p=0.0785, ir64a: p=0.7151, or92a: p=0.6639, or42b: p=0.1488) for any genotype.

E) Timecourse of upwind velocity and curvature for flies with orco+ ORNs silenced using orco>TNT (N= 25 flies, teal) versus UAS-TNT controls (N= 31 flies, gray).

All timecourses show mean +/-SEM. All quantifications show mean+/-STD overlaid. All statistics use two-sided Wilcoxon sign rank test and are Bonferroni corrected for multiple comparisons.

Supplementary Figure 2

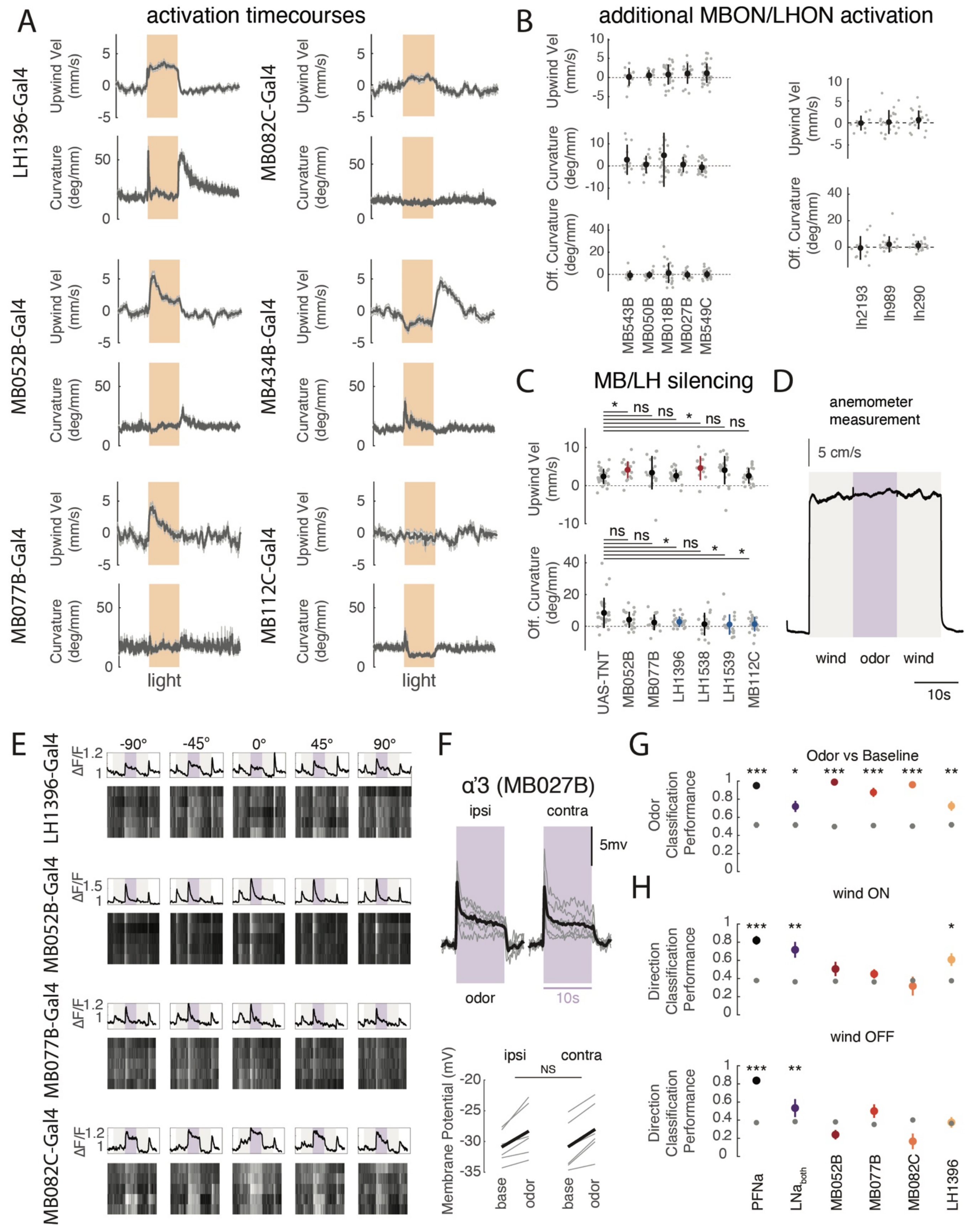

Fig. S2: Additional data on LH and MB responses and behavior

A) Upwind velocity and curvature time courses (mean±SEM) for LH1396>Chrimson (N=24 flies), MB052B>Chrimson (N=27), MB077B>Chrimson (N=21), MB082C>Chrimson (N=24), MB0434B>Chrimson (N=24), and MB112C>Chrimson (N=29).

B) Upwind velocity, curvature during stimulus, and OFF curvature for individual MBONs labeled by MB052B (left) and for additional LHONs (right). MBONs: upwind: MB543B (N=12): p=0.5693, MB050B (N=14): p=0.1726, MB018B (N=27): p=0.1363, MB027B (N=16): p=0.1477, MB549C (N=35): p=0.0360, curvature: MB110C: p=0.0027, MB543B: p=0.3804, MB083C: p=3.9023e-04, MB050B: p=0.5016, MB018B: p=0.2029, MB027B: p=0.5695, MB549C: p=0.0742 OFF curvature: MB543B: p=0.2061, MB050B: p=0.3910, MB018B: p=0.7533, MB027B: p=0.4380, MB549C: p=0.7807. LHONs: upwind: LH2193 (N=11): p=0.9658, LH989(N=22): p=0.9095, LH290(N=24): p=0.0975, OFF curvature: LH2193: p=0.7002, LH989: p=0.1997, LH290: p=0.1443.

C) Effects of MBON and LHON silencing on upwind velocity and OFF curvature, compared to UAS-TNT control(N=31). Upwind velocity: MB052B (N=23): p=0.0036862, MB077B (N=17): p=0.17444, LH1396 (N=24): p=0.6529, LH1538 (N=16): p=0.0036449, LH1539 (N=21): p=0.0090304, MB112C (N=22): p=0.83555; OFF curvature: MB052B: p=0.063702, MB077B: p=0.012398, LH1396: p=0.0027408, LH1538: p=0.013958, LH1539: p=0.00070953, MB112C: p=0.0011212.

D) Average stimulus delivered to the fly from 3 trials from each of the 5 directions as measured by a hotwire anemometer (average across directions). There is no change in windspeed between wind alone and wind + odor.

E) Single fly examples of calcium responses to wind and odor in LH1396, MB052B, MB077B, and MB082C. Top boxes show average traces for single flies across 5 trials from each direction. Heat maps below depict responses for individual trials.

F) Membrane potential responses in α'3 MBONs (labeled by MB027B) to 10% vinegar presented from 90° ipsilateral or contralateral to the recorded neuron. Black trace represents mean across flies while gray traces represent individual flies (N=6 cells per hemisphere, each from 1 fly). Odor significantly increases membrane voltage both ipsilaterally and contralaterally (paired two-sided student t-test p= 0.0018, 0.0159) and is not different between sides (unpaired two-sided student t-test p=0.7561).

G) Performance of a tree classifier at decoding odor versus baseline activity, trained on 5s of baseline versus first 5s of odor ON. Gray dots represent a classifier trained with the same data and shuffled labels. PFNa (N=11)p= 3.2874e-10, LNa (N=5) p= 0.0040, MB052B (N=9) p= 7.9669e-18, MB077B (N=8) p= 7.7200e-07, MB082C (N=5) p= 2.3966e-12, LH1396 (N=8) p= 0.0010.

H) Performance of tree classifiers at decoding wind direction (left, center, right). Top: classifier trained on the first 5s of wind ON. PFNa (N=11) p= 2.3499e-08 LNa (N=5) p= 9.0101e-04 , MB052B(N=9) p= 0.1095 , MB077B (N=8) p= 0.1122, MB082C (N=5) p= 0.5650 , LH1396 (N=8) p= 0.0039. Bottom: trained on the 5s following wind OFF. Student's t-test, PFNa p= 2.5701e-14, LNa p= 0.1572, MB052B p= 0.0161, MB077B p= 0.0670, MB082C p= 0.0157, LH1396 p= 0.8713. Gray dots represent classifiers trained with the same data and shuffled labels.

Statistics in **B** use two-sided Wilcoxon signed rank test and show mean+/- STD overlaid. Statistics in **C** use two-sided Mann Whitney U test compared to UAS-TNT control and show mean+/- STD overlaid. Classifiers in **G,H** used two-sided student's t-tests and show mean±SEM. All statistics corrected using the Bonferroni method.

Supplementary Figure 3

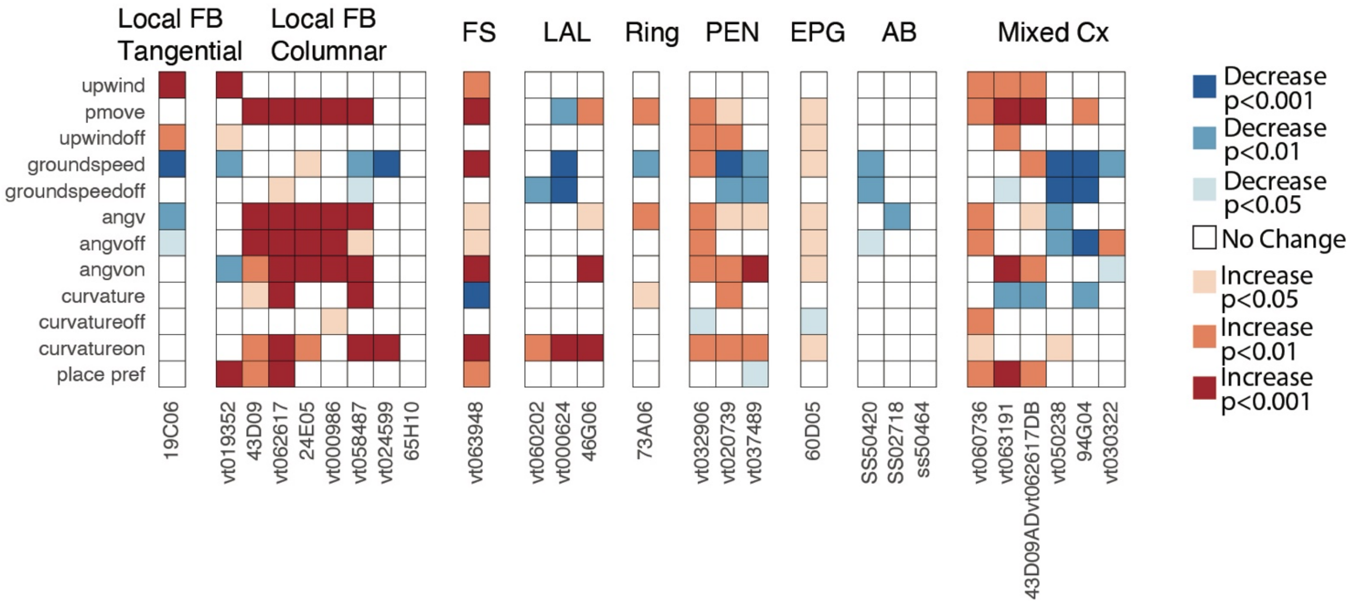

Fig. S3: Additional data from optogenetic screen of CX neurons

Optogenetic activation data for additional central complex neuron types: local FB tangential neurons, local FB columnar neurons, FS neurons, LAL innervating neurons, ring neurons, PEN neurons, EPG neurons, AB neurons, and drivers that labelled more than one CX type prominently (mixed). One local tangential FB neuron line (19C06), one local columnar FB neuron line (VT019352), and three mixed CX lines (VT060736, VT063191, the split line 43D09-AD; VT062617-DB) and one FS neuron line (VT063948) produced significant increases in upwind velocity. No LAL, Ring, PEN, or AB lines produced significant changes in upwind velocity, nor did activation of EPG compass neurons (60D05). Several FB local columnar neuron lines drove increases in movement probability (Pmove) and angular velocity (angv): 43D09, VT062617, 24E05, VT000986, VT058487. See Methods for calculation of behavioral parameters. All comparisons used two-sided Wilcoxon signed rank tests and are Bonferroni corrected for multiple comparisons.

Supplementary Figure 4

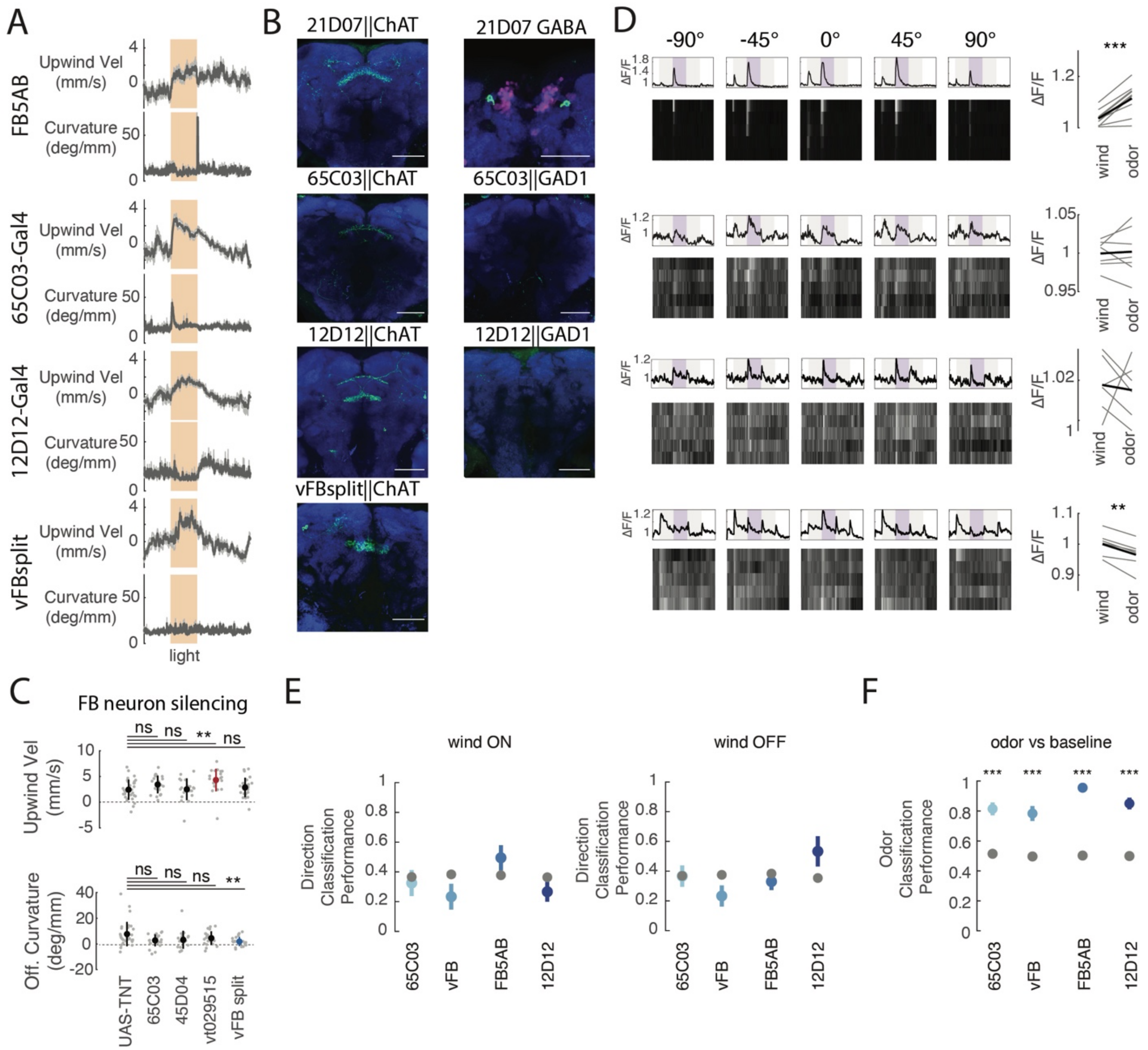

Fig. S4: Additional data on odor-responsive, upwind-promoting FB tangential neurons

A) Timecourses of upwind velocity and curvature averaged across flies for all FB tangential input lines that showed significant responses to vinegar (mean±SEM), 21D07IICLIN (N=27), 65C03 (N=24), 12D12 (N=38), vFB split (N=27)).

B) Odor-responsive FB tangential inputs are cholinergic and not GABAergic. Left column: Flies expressing *Chrimson-mVenus* under the control of each driver using a ChAT-LexA,LexAOP-FLP strategy (green, see Methods). Right column: 21D07: same genotype as left, but co-stained with anti-GABA (magenta). There is no colocalization of GABA and Chrimson-mVenus. Flies expressing *Chrimson-mVenus* using a Gad1-LexA, LexAOP-FLP strategy (green) do not label FB neurons in 65C03 or 12D12. Scale bars represent 50μM.

C) Effects of FB neuron silencing on upwind velocity and OFF curvature, compared to UAS-TNT control(N=31). Upwind: 65C03 (N=24): p=0.048027, 45D04 (N=20): p=0.65028, vt029515 (N=22): p=0.00086718, vFB split (N=24) p=0.60472, OFF curvature: 65C03: p=0.044317, 45D04: p=0.087747, vt029515: p=0.31643, vFB split: p=0.0021892.
D) Left: Example calcium responses from FB tangential inputs in individual flies. Top row depicts average ΔF/F for each direction across 5 trials, heat maps below depict responses in individual trials. Right: Calcium response to wind alone vs vinegar. Gray lines represent individual flies and black lines represent genotype means. Significant increase for 21D07 (p=3.9090e-04). No significant increase for 65C03 or 12D12 (p=0.7790, p=0.7782) and significant decreases for vFB split (p = 0.0028)
E) Performance of tree classifiers at decoding wind direction (left, center, right). Left: classifier trained on the first 5s of wind ON. Student's t-test: 65C03 (N=7) p=0.6504, vFB (N=6) p=0.1088, FB5AB (N=9) p=0.1859, 12D12 (N=6) p=0.1650. Right: classifier trained on the 5s following wind OFF. 65C03 p=0.9802, vFB p=0.0646, FB5AB p=0.3656, 12D12 p=0.0995. Due to stimulus presentation differences between preps, PFNa data here is shown relative to wind ON as a control. Gray dots represent classifiers trained with the same data and shuffled labels.
F) Performance of a tree classifier at decoding odor versus baseline activity, trained on 5s of baseline versus first 5s of odor ON. Gray dots represent a classifier trained with the same data and shuffled labels. 65C03 (N=7) p=1.6787e-06, vFB (N=6) p=2.0903e-05, FB5AB (N=9) p=5.7008e-13, 12D12 (N=6) p=5.8079e-08.
Statistics in **C** use two-sided Mann Whitney U test compared to UAS-TNT control and show mean+/-STD overlaid. Statistics in **D** use paired two-sided student t-test. Classifiers in **E,F** used two-sided student's t-tests and show mean±SEM. All statistics corrected using the Bonferroni method.

Supplementary Figure 5

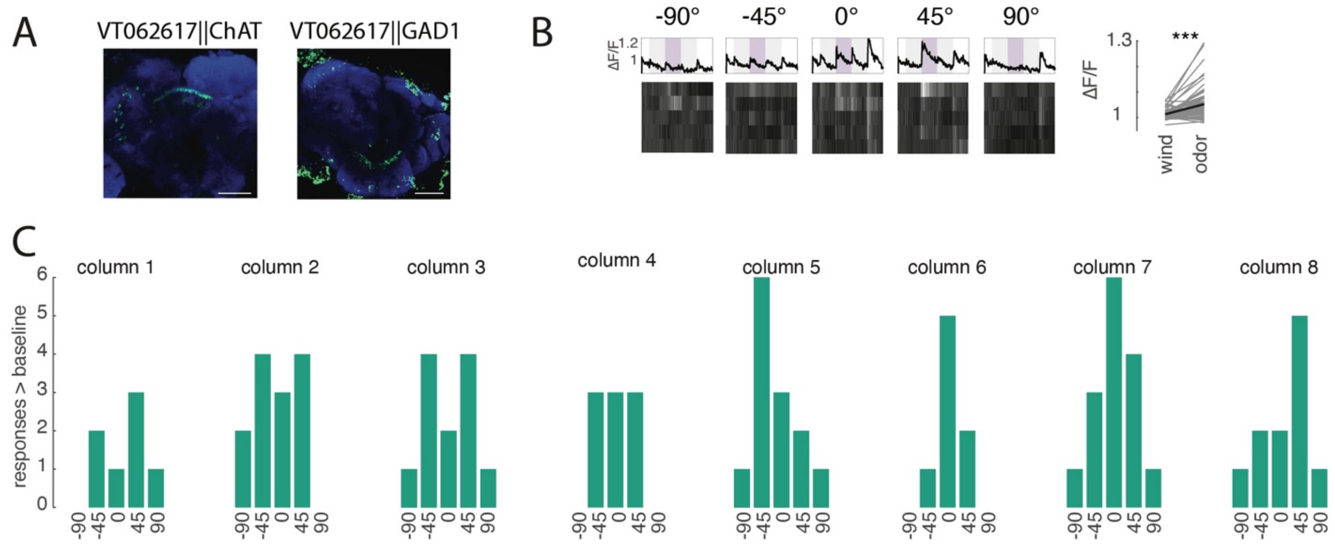

Fig. S5: Additional data on hΔC responses

A) hΔC neurons are cholinergic. Left: flies expressing *Chrimson-mVenus* under the control of VT062617-GAL4 using a ChAT-LexA,LexAOP-FLP strategy (green, see Methods). Right: flies expressing *Chrimson-mVenus* using a Gad1-LexA, LexAOP-FLP strategy (green) do not label FB neurons in VT062617-GAL4. Scale bars represent 50μM.

B) Calcium response examples from an individual fly for hΔC (left). Top row depicts average ΔF/F for each direction across 5 trials, heat maps below depict responses in individual trials. Right: Calcium response to wind alone (5s after wind ON) vs vinegar (5s after odor ON). Gray lines represent average increases of individual active columns. Odor response is significantly larger than wind response (paired two-sided student t-test: 4.7885e-11).

C) Directional responses are not correlated with anatomical column. Histograms depict counts of directional responses during the odor period (first 5s) for each column >2STD above baseline period. Columns 1-8 correspond to fly's right to left.

Supplementary Figure 6

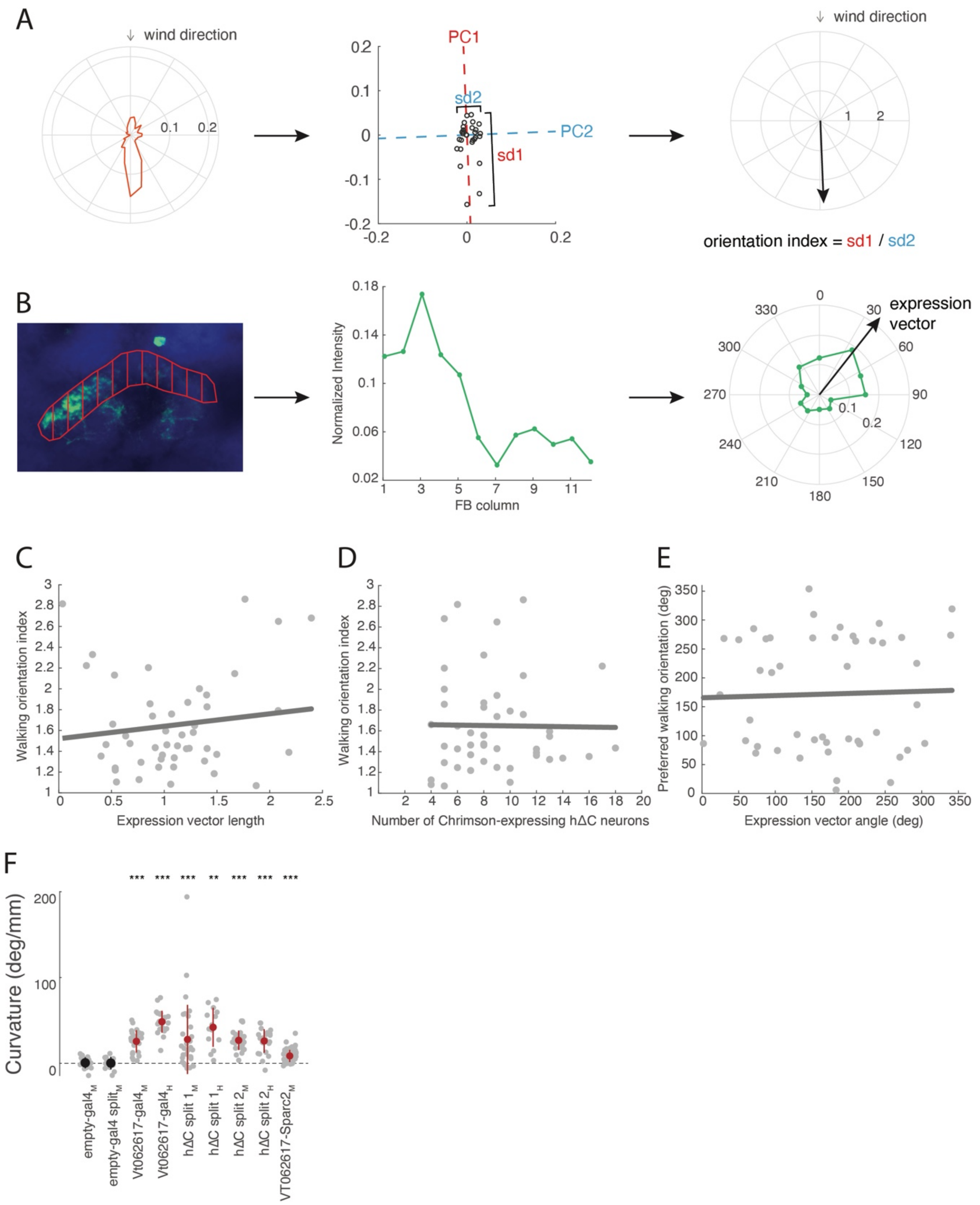

Fig. S6: Additional data on hΔC activation

A) Strategy for calculating the walking orientation index and preferred walking direction of SPARC flies. Orientation data from 2-6 s after light ON were converted into an orientation histogram (left). We performed PCA on this histogram in Cartesian coordinates (center), then computed the orientation index as the ratio of the standard deviation along PC1 to the standard deviation along PC2 (right). We computed the preferred walking direction as the direction of PC1.

B) Strategy for calculating the expression vector of hΔC > SPARC flies. We divided the output tufts of hΔC neurons into 12 columns (left), and computed normalized fluorescence across these columns (middle). We then converted the normalized expression profile into polar coordinates and summed to create an expression vector (right).

C) The orientation index of hΔC > SPARC flies is not related to the expression vector length (correlation: $p = 0.3607$, $r^2 = 0.1349$).

D) The orientation index of hΔC > SPARC flies is not related to the total number of Chrimson-expressing hΔC neurons (correlation: $p = 0.9257$, $r^2 = 0.0002$).

E) The preferred direction of hΔC > SPARC flies is not related to the expression vector angle (correlation: $p = 0.8263$, $r^2 = 0.0011$).

F) Curvature during optogenetic activation (mean ± STD) of empty GAL4 and various hΔC lines with medium (M: 26 μW/mm^2) or high (H: 34 μW/mm^2) light power. Empty-Gal4 (N=19): p=0.5732, empty split (N=14) p=0.7869, VT062617-Gal4 (N=25) (medium) p= 1.2290e-05, VT062617-Gal4 (N=25) (high) p=1.2290e-05, hΔC split1 (N=32) (medium) p=1.8517e-05, hΔC split1 (N=14) (high) p=1.2207e-04, hΔC split2 (N=27) (medium) p=5.6061e-06, hΔc split2 (N=24) (high) p=2.3518e-05, VT062617-SPARC2 (N=71) (medium) p=2.2783e-12. All statistics use twosided Wilcoxon signed rank test and are Bonferroni corrected for multiple comparisons.

Supplementary Figure 7

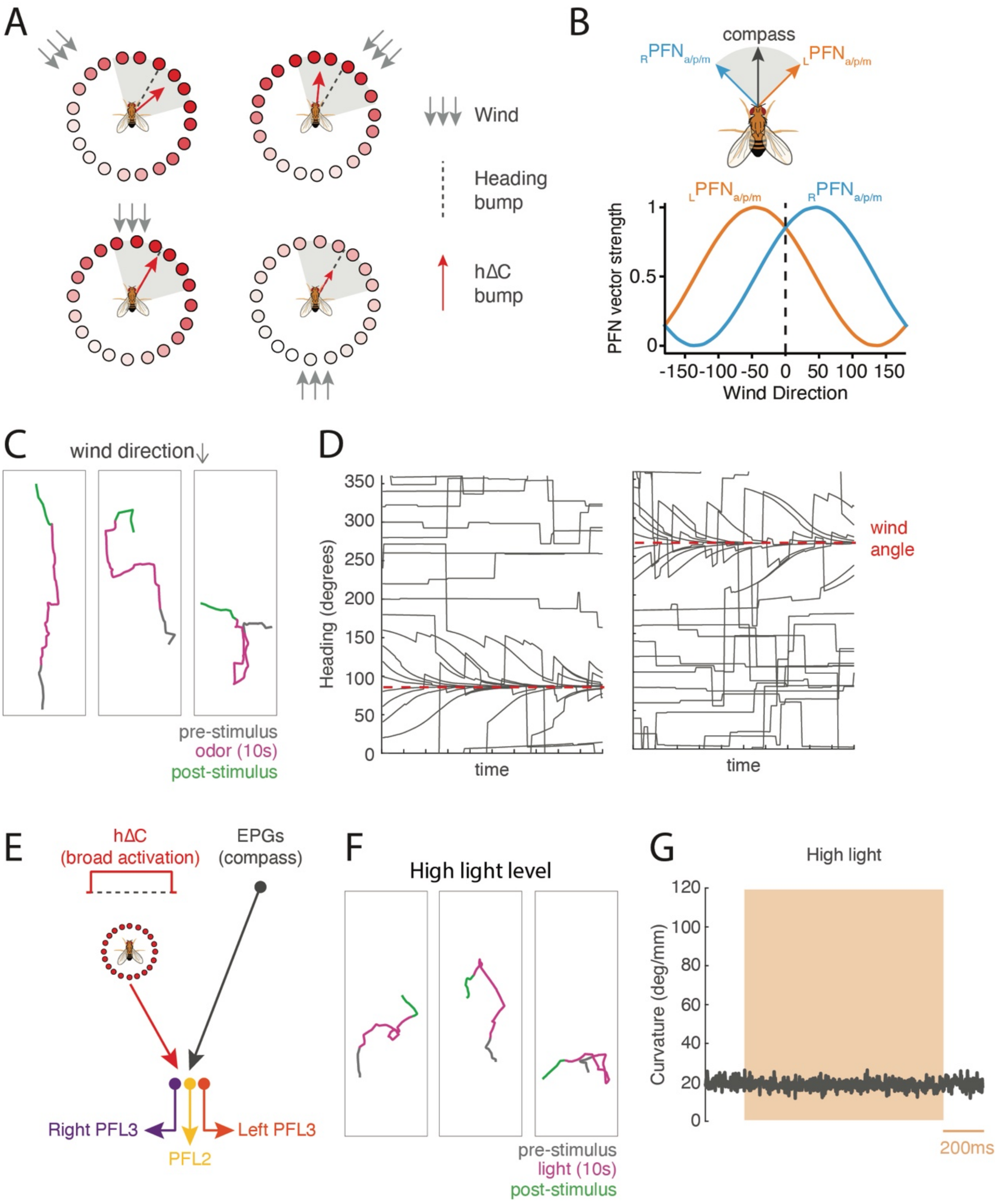

Fig. S7: Additional model simulations

A) Alternate model wind representation in hΔC neurons, that can be built from two frontally tuned PFN populations as described in Currier et al. 2020. Wind direction is represented as a bump of activity (circles) and as a vector (red), while heading direction is represented as a vector (gray). As in the allocentric representation (Fig. 7B), the wind vector is to the right of the heading vector for leftward wind and to the left of the heading vector for rightward wind. However, in this scheme, wind vectors are contained within a 90° window centered around the heading vector, and wind from the rear produces a bump of reduced amplitude.
B) Hypothesized wind representation in wind-tuned PFNs (PFNa/p/m) based on physiology from Currier et al. 2020 and anatomy from Hulse et al. 2021. PFNs receive a shifted heading bump from the compass such that left and right hemisphere PFNs exhibit bumps offset by 45° for left PFNs and -45° for right PFNs (top). PFNs in each hemisphere are maximally activated by wind arriving from 45° ipsilateral (-45° for left PFNs, +45° for right PFNs) such that the length of the vectors increase or decrease across wind directions (bottom).
C) Behavioral trajectories of simulated flies using the frontal wind representation in **A** before, during, and after odor. Note that flies go upwind so long as they are initially orientated within +/- 90° of upwind.
D) Heading of simulated flies using the frontal wind representation in **A** for two different wind directions and several different initial headings. Note that flies go upwind so long as they are initially orientated within +/- 90° of upwind, or reach one of these orientations through random turns.
E) Simulated circuit activity during broad optogenetic activation without mutual inhibition. In this simulation, every hΔC neuron is activated equally for the duration of the stimulus but the mutual inhibition layer is omitted.
F) Behavioral trajectories of simulated flies lacking the mutual inhibition circuit during broad hΔC activation. Random turn rate is the same before, during, and after stimulation.
G) Average curvature simulated flies lacking the mutual inhibition circuit during broad optogenetic activation using high light. Without the mutual inhibition circuit, broad activation of hΔC neurons does not drive an increase in curvature.

Table S1: Genotype and age information for all figure panels

Genotype	Description	Age range	Figure panels
**Figure 1, S1**			
norpA$_{36}$/y;UAS-Chrimson-mVenus/orco-GAL4;UAS-10xGFP/+	genetically blind male flies expressing Chrimson under the orco promoter	3-10 days	vinegar experiments in Fig. 1C,D,E Fig S1A
norpA$_{36}$/y;tsh-gal80/orco-GAL4,IR8a-GAL4;UAS-Chrimson-mVenus/+	genetically blind male flies expressing Chrimson under the orco and IR8a promoters	3-10 days	Optogenetic activation experiments in Fig. 1C,D,E Fig. S1C
norpA$_{36}$/y;UAS-Chrimson-mVenus;UAS-10xGFP x X-GAL4 or split-GAL4<br><br>X-GAL4: orco-GAL4,IR8a-GAL4 (II), empty-GAL4 (II), empty split-GAL4 (II,III), OR59a (III), OR42a (III), IR75a (III), IR64a (II), OR92A (III), OR42b (II)	genetically blind hemizygous male flies expressing Chrimson under various GAL4 or split-GAL4 drivers on chromosomes II or III.	3-10 days	optogenetic activation experiments in Fig. 1E Fig. S1A,B,D
norpA$_{36}$/y;+/UAS-Chrimson-mVenus;+/UAS-10xGFP	parental control	3-10 days	parental control for activation in Fig. 1E
UAS-TNT$_{5905}$	parental control; UAS-TNT backcrossed 5 generations to w1118 5905	3-10 days	parental control for silencing in Fig. 1F
UAS-TNT$_{5905}$ x X-GAL4<br><br>X-GAL4: orco-GAL4, IR8a-GAL4, orco-GAL4,IR8a-GAL4	flies with various ORNs silenced	3-10 days	Fig. 1F,G, Fig. S1E
**Figure 2, S2**			
norpA$_{36}$/y;tsh-Gal80;UAS-Chrimson-mVenus x X-GAL4 or split-GAL4<br><br>X-GAL4/split-GAL4: LH1538, LH1396, LH1539, MB082C, MB077B, MB052B, MB434B, MB112C, MB011B, MB543B, MB050B, MB018B, MB027B, MB549C, LH2193, LH989, LH290.	genetically blind hemizygous male flies expressing Chrimson under various GAL4 or split-GAL4 drivers on chromosomes II or III. tsh-Gal80 included to suppress VNC expression.	3-10 days	Fig. 2A-D, Fig. S2A,B
UAS-TNT$_{5905}$ x X-GAL4 or split-GAL4	flies with MB/LH neurons silenced	3-10 days	Fig. S1C

X-GAL4: LH1538, LH1396, LH1539, MB077B, MB052B, MB112C or + for parental control			
+;UAS-GCaMP6f;UAS-tdTomato x X-GAL4<br><br>X-GAL4: LH1396, MB052B, MB077B, MB082C, ss47432 (LNa)	GCaMP6f and tdTOM expressed in LHAd1b2 using the LH1396 split GAL4 driver or in MBONs15-19 using MB052B, MBON12 using MB077B, or MBON 13,14 using MB082C, LNa using ss47432	5-21 days	Fig. 2E, Fig. S2E
+;MB027B-GAL4AD/+;UAS-10xGFP/MB027B-GAL4DB	expression of GFP in ’3 MBONs for electrophysiology	1-3 days	Fig. 2F
**Figure 3, S3**			
UAS-myrGFP, QUAS-mtdTomato (3x HA); trans-Tango x X-GAL4<br><br>X-GAL4: LH1396, MB052B	Trans-tango driven by LH1396 and MB052B	10-20 days; raised at 19°C	Fig. 3A
norpA^{36}/y;tsh-Gal80;UAS-Chrimson-mVenus x X-GAL4 or split-GAL4<br><br>Dorsal Inputs: VT056792-GAL4, VT002458-GAL4, 84C10-GAL4, VT027955-GAL4, 58F01-GAL4, 58F02-GAL4, 71A02-GAL4,VT026663-GAL4, VT20633-GAL4, VT004849-GAL4, 23E10-GAL4, 26B11-GAL4, 28H10-GAL4, 12D12-GAL4, 65C03-GAL4, 45D04-GAL4, LH2392<br><br>Ventral Inputs: VT036875-GAL4, 33E06-GAL4, VT056509-GAL4, 72A04-GAL4, 78G09-GAL4, vt049652-GAL4, 13B10-GAL4, VT033929-GAL4, VT046276-GAL4, VT041421-GAL4,VT029515-GAL4<br><br>PFN: 44B10-GAL4; VT039497-GAL4,16D01-GAL4, 67B06-	genetically blind hemizygous male flies expressing Chrimson under various GAL4 or split-GAL4 drivers on chromosomes II or III. tsh-Gal80 included to suppress VNC expression.	3-10 days	Fig. 3B, C, S3

GAL4, SS52577, SS52244, SS54549, SS02255, SS00239, 43D09AD; VT000986DB<br><br>Split-GAL4: 43D09-AD;65C03-DB, 12D12-AD;VT027955-DB, VT026663AD;65C03DB, 12D12AD;65C03DB, VT041421AD;28H10DB, VT041421AD;33E06DB, VT041421DB;65H10DB, VT029515AD; 78G09DB, LH1478, 13B10AD;VT029515DB, 13B10AD; VT041421DB<br><br>Blank: Empty-GAL4, Empty Split-Gal4			
norpA^{36}/y;tsh-Gal80;UAS-Chrimson-mVenus x X-GAL4 or split-GAL4<br><br>Additional CX neurons: 19C06-GAL4, 65H10-GAL4, VT024599-GAL4, VT058487-GAL4, VT000986-GAL4, 24E05-GAL4, VT062617-GAL4, 43D09-GAL4, VT019352-GAL4, VT030322-GAL4, 94G02-GAL4, VT050238-GAL4, VT063191-GAL4, VT060736-GAL4, 43D09AD;VT062617DB, VT063948-GAL4, 46G06-GAL4, V000624-GAL4, VT060202-GAL4, 73A06-GAL4, VT037489-GAL4, VT020739-GAL4, VT032906-GAL4, 60D05-GAL4, SS50464, SS02718, SS50420			
**Figure 4, S4**			
CLIN x UAS-Chrimson-mVenus;21D07-GAL4	21D07>Chrimson-mVenus filtered through CLIN to restrict expression to type II (CX) neurons	3-10 days	Fig. 4A (anatomy),C (behaviour)<br>Fig. S4A
+;UAS-GCaMP6f;UAS-tdTomato x X-GAL4	GCaMP6f and tdTOM expressed under the various FB drivers	5-21 days	Fig. 4B,D imaging, Fig. S4D,E

X-GAL4: 21D07-GAL4, 65C03-GAL4, vFB split (13B10AD;VT041421DB), 12D12-GAL4			
+;UAS-GCaMP6f;UAS-tdTomato x X-GAL4<br><br>X-GAL4: MB052B, MB077B, MB082C, LH1396, 21D07-GAL4, 65C03-GAL4, vFB split (13B10AD;VT041421DB), 12D12-GAL4	GCaMP6f and tdTOM expressed under the various drivers	5-21 days	Fig. 4E
$norpA_{36}$/y;tsh-Gal80;UAS-Chrimson-mVenus x X-GAL4 or split-GAL4<br><br>X-GAL4: 65C03-GAL4, 12D12-GAL4, vFB split (13B10AD;VT041421DB), VT029515-GAL4	genetically blind hemizygous male flies expressing Chrimson under various GAL4 or split-GAL4 drivers on chromosomes II or III. tsh-Gal80 included to suppress VNC expression.		Fig. 4A (anatomy),C (behavior), Fig. S4A
UAS(FRT.stop)-Chrimson-mVenus; LexAop-FLP; ChAT-LexA; ChAT-LexA x X-GAL4 or split-GAL4<br><br>X-GAL4: 21D07-GAL4, 65C03-GAL4, vFB split (13B10AD;VT041421DB), 12D12-GAL4	cross to identify cholinergic neurons within a GAL4 or split-GAL4 line	3-20 days	Fig. S4B
UAS(FRT.stop)-Chrimson-mVenus; LexAop-FLP; Gad1-LexA x X-GAL4 or split-GAL4<br><br>X-GAL4: 65C03-GAL4, 12D12-GAL4	cross to identify GABAergic neurons within a GAL4 or split-GAL4 line	3-20 days	Fig. S4B
UAS-TNT_{5905} x X-GAL4 or split-GAL4<br><br>X-GAL4: 65C03-GAL4, 45D04-GAL4, VT029515-GAL4, vFB split (13B10AD;VT041421DB)	flies with FB neurons constitutively silenced	3-10 days	Fig. S4C
**Figure 5, S5**			
+;UAS-GCaMP6f;UAS-tdTomato x VT062617-GAL4	GCaMP6f and tdTOM expressed in hΔC neurons	5-21 days	Fig. 5D-H, S5A
UAS(FRT.stop)-Chrimson-mVenus; LexAop-FLP; ChAT-	cross to identify cholinergic neurons within a GAL4 line	3-20 days	Fig. S5A

LexA; ChAT-LexA x VT062617-GAL4			
UAS(FRT.stop)-Chrimson-mVenus; LexAop-FLP; Gad1-LexA x VT062617-GAL4	cross to identify GABAergic neurons within a GAL4 line	3-20 days	Fig. S5A
**Figure 6, S6**			
20xUAS-SPARC2-I-Syn21-CsChrimson::tdTomato x<br><br>UAS-phiC31;VT062617-GAL4<br>UAS-phiC31;empty-GAL4	15% expression of Chrimson using SPARC2-I in hΔC neurons or empty-GAL4	3-7 days	Fig. 6 A-E, S6A-B
norpA^{36}/y;tsh-Gal80;UAS-Chrimson-mVenus x X-GAL4 or split-GAL4<br><br>X-GAL4: VT062617-GAL4, hΔC split1 (19G02AD;VT062617DB), hΔC split2 (VT024634AD; VT062617DB)	genetically blind hemizygous male flies expressing Chrimson under various GAL4 or split-GAL4 drivers on chromosomes II or III. tsh-Gal80 included to suppress VNC expression.	3-10 days	Fig. 6D-F
w^{1118}/+;s/+;X-GAL4/UAS-GtACR1 or<br>w^{1118}/+;s/X-GAL4; UAS-GtACR1/+<br>X-GAL4(II): orco-GAL4, IR8a-GAL4X-GAL4(III): 21D07-GAL4, VT062617-GAL4	Acute silencing of ORNs, FB5AB, and hΔC with GtACR, in genetically blind hemizygous males	3-10 days	Fig. 6G-H

Table S2: List of parental stocks and identifiers:

Stock	Source	Identifier
D. mel: orco-GAL4 (backcrossed into *w118* 5905)	BDSC	RRID:BDSC_26818
D. mel: OR59a-GAL4 (III)	BDSC via Marc Gershow	RRID:BDSC_9990
D. mel: OR42b-GAL4 (II)	BDSC via Marc Gershow	RRID:BDSC_9971
D. mel: OR42a-GAL4 (III)	BDSC	RRID:BDSC_9969
D. mel: IR75a-GAL4/TM6B	BDSC	RRID:BDSC_41748
D. mel: IR64a-GAL4;TM2/TM6	BDSC	RRID:BDSC_41732
D. mel: OR92a-GAL4	BDSC	RRID:BDSC_23139
D. mel: IR8a-GAL4;TM2/TM6	BDSC	RRID:BDSC_41731
D. mel: 60D05-GAL4	BDSC	RRID:BDSC_39247
D. mel: 46G06-GAL4	BDSC	RRID:BDSC_41271
D. mel: 44B10-GAL4	BDSC	RRID:BDSC_50202
D. mel: 16D01-GAL4	BDSC	RRID:BDSC_48722
D. mel: 19C06-GAL4	BDSC	RRID:BDSC_48843
D. mel: 67B06-GAL4/TM3	BDSC	RRID:BDSC_48294
D. mel: 73A06-GAL4	BDSC	RRID:BDSC_39805
D. mel: 84C10-GAL4	BDSC	RRID:BDSC_48378
D. mel: 71A02-GAL4	BDSC	RRID:BDSC_39560
D. mel: 23E10-GAL4	BDSC	RRID:BDSC_49032
D. mel: 26B11-GAL4	BDSC	RRID:BDSC_49164
D. mel: 28H10-GAL4	BDSC	RRID:BDSC_48085
D. mel: 12D12-GAL4	BDSC	RRID:BDSC_48506
D. mel: 65C03-GAL4	BDSC	RRID:BDSC_41290
D. mel: 45D04-GAL4	BDSC	RRID:BDSC_48160
D. mel: 65H10-GAL4	BDSC	RRID:BDSC_49614
D. mel: 43D09-GAL4	BDSC	RRID:BDSC_49553
D. mel: 24E05-GAL4	BDSC	RRID:BDSC_49081
D. mel: 94G04-GAL4	BDSC	RRID:BDSC_40699
D. mel: 33E06-GAL4	BDSC	RRID:BDSC_48114
D. mel: 72A04-GAL4	BDSC	RRID:BDSC_46665
D. mel: 78G09-GAL4	BDSC	RRID:BDSC_40015
D. mel: 13B10-GAL4	BDSC	RRID:BDSC_48548
D. mel: 21D07-GAL4	BDSC	RRID:BDSC_48943
D. mel: 58F01-GAL4	BDSC	RRID:BDSC_48213
D. mel: 58F02-GAL4	BDSC	RRID:BDSC_39186
D. mel: empty GAL4	BDSC	RRID:BDSC_68384
D. mel: empty split GAL4	BDSC	RRID:BDSC_79603
D. mel: 43D09AD	BDSC	RRID:BDSC_70691
D. mel: VT000986DB	BDSC	RRID:BDSC_75369
D. mel: VT026663AD	BDSC	RRID:BDSC_73054
D. mel: 12D12DB	BDSC	RRID:BDSC_69213

D. mel: VT027955DB	BDSC	RRID:BDSC_73229
D. mel: 65C03DB	BDSC	RRID:BDSC_69323
D. mel: 65C03AD	BDSC	RRID:BDSC_71005
D. mel: 45D04-DB	BDSC	RRID:BDSC_69239
D. mel: 12D12AD	BDSC	RRID:BDSC_70539
D. mel: VT041421AD	BDSC	RRID:BDSC_71457
D. mel: 28H10DB	BDSC	RRID:BDSC_69496
D. mel: 33E06DB	BDSC	RRID:BDSC_69229
D. mel: 65H10DB	BDSC	RRID:BDSC_86702
D. mel: VT029515AD	BDSC	RRID:BDSC_74286
D. mel: 78G09DB	BDSC	RRID:BDSC_69718
D. mel: 13B10AD	BDSC	RRID:BDSC_68828
D. mel: VT029515DB	BDSC	RRID:BDSC_75470
D. mel: VT041421DB	BDSC	RRID:BDSC_73472
D. mel: VT063948-GAL4	VDRC	VDRC: 200657
D. mel: VT000624-GAL4	VDRC	VDRC: 202720
D. mel: VT060202-GAL4	VDRC	VDRC: 202779
D. mel: VT037489-GAL4	VDRC	VDRC: 206278
D. mel: VT020739-GAL4	VDRC	VDRC: 201501
D. mel: VT032906-GAL4	VDRC	VDRC: 202537
D. mel: VT039497-GAL4	VDRC	VDRC: 205730
D. mel: VT056792-GAL4	VDRC	VDRC: 206056
D. mel: VT002458-GAL4	VDRC	VDRC: 202329
*D. mel: VT*027955-GAL4	VDRC	VDRC: 202476
D. mel: VT026663-GAL4	VDRC	VDRC: 203208
D. mel: VT020633-GAL4	VDRC	VDRC: 207839
D. mel: VT004849-GAL4	VDRC	VDRC: 207295
D. mel: VT024599-GAL4	VDRC	VDRC: 202132
D. mel: VT058487-GAL4	VDRC	VDRC: 207739
D. mel: VT062617-GAL4	VDRC	VDRC: 206875
D. mel: VT000986-GAL4	VDRC	VDRC: 207036
D. mel: VT019352-GAL4	VDRC	VDRC: 201718
D. mel: VT030322	VDRC	VDRC: 204795
D. mel: VT050238-GAL4	VDRC	VDRC: 201475
D. mel: VT063191-GAL4	VDRC	VDRC: 205643
D. mel: VT060736-GAL4	VDRC	VDRC: 202781
D. mel: VT036875-GAL4	VDRC	VDRC: 203402
D. mel: VT056509-GAL4	VDRC	VDRC: 201744
D. mel: VT049652-GAL4	VDRC	VDRC: 204721
D. mel: VT033929-GAL4	VDRC	VDRC: 206842
D. mel: VT046276-GAL4	VDRC	VDRC: 207814
D. mel: VT041421-GAL4	VDRC	VDRC: 200867
D. mel: VT029515-GAL4	VDRC	VDRC: 204785
D. mel: LH1396	Janelia	Janelia: 3026621
D. mel: LH1538	Janelia	Janelia: 3024098
D. mel: LH1539	Janelia	Janelia: 3024933

D. mel: LH989	Janelia	Janelia: 3032170
D. mel: LH290	Janelia	Janelia: 2502068
D. mel: LH2392/CyO;TM6	Janelia	Janelia: 3024051
D. mel: LH1478/CyO;TM6	Janelia	Janelia: 3032196
D. mel: LH2193	Janelia	Janelia: 3024119
D. mel: MB052B	Karla Kaun	
D. mel: MB077B	Karla Kaun	Janelia: 2135209
D. mel: MB543B	Karla Kaun	Janelia: 2501888
D. mel: MB050B0	Karla Kaun	Janelia: 2135100
D. mel: MB018B/CyO	Karla Kaun	Janelia: 2135069
D. mel: MB027B	Karla Kaun	Janelia: 2135078
D. mel: MB549C/TM6b	Karla Kaun	Janelia: 2501897
D. mel: MB082C	Karla Kaun	Janelia: 2135130
D. mel: MB434B/CyO	Janelia	Janelia: 2501775
D. mel: MB112C	Janelia	Janelia: 2135209
D. mel: MB011B	Janelia	Janelia: 2135062
D. mel: SS52577	Janelia	Janelia: 3028433
D. mel: SS02255/[TM6b]	Janelia	Janelia: 3018121
D. mel: SS52244/[CyO]	Janelia	Janelia: 3028329
D. mel: SS54549	Janelia	Janelia: 3029433
D. mel: SS02239	Janelia	Janelia: 3018105
D. mel: SS50464	Janelia	Janelia: 3028081
D. mel: SS02718	Janelia	Janelia: 3018714
D. mel: SS50420	Janelia	Janelia: 3028108
D. mel: SS47432	Janelia	Janelia: 3027368
D. mel: orco-GAL4,IR8a-GAL4/[CyO]	this study	
D. mel: 43D09AD;VT000986DB	this study	
D. mel: VT026663AD;12D12DB	this study	
D. mel: VT026663AD;VT027955DB	this study	
D. mel: 43D09AD;65C03DB	this study	
D. mel: 65C03AD;45D04DB	this study	
D. mel: 12D12AD;vt027955DB	this study	
D. mel: VT026663AD;65C03DB	this study	
D. mel: 12D12AD;65C03DB	this study	
D. mel: VT041421AD;28H10DB	this study	
D. mel: VT041421AD;33E06DB	this study	
D. mel: VT041421AD;65H10DB	this study	
D. mel: VT029515AD;78G09DB	this study	
D. mel: 13B10AD;VT029515DB	this study	
D. mel: 13B10AD;VT041421DB	this study	
D. mel: 20XUAS-IVSCsChrimson.mVenus}attP40 (II)	BDSC	RRID: BDSC_55135

D. mel: 20x-UAS-IVS-csChrimson-mVenus-attp2 (III)	BDSC	RRID: BDSC_55136
D. mel: UAS-tdTOM (III)	BDSC	RRID: BDSC_36328
D. mel: norpA^36;T(2,3)En^es/([cyo-afgp;TM3-agfp])	Rachel Wilson	
D. mel: +(HCS);+;P{10xUAS-IVS-Syn21-GFP-p10}attP2 (10X UAS- GFP) (Backcrossed)	Michael Dickinson	
D. mel: UAS-GCaMP6f;UAS-tdTOM	Michael Dickinson	
D. mel: w[*]; P{w[+mC] = UAS-TeTxLC.tnt}E2 (UAS-TNTe)	Matthieu Louis	
tsh-Gal80(w-)/CyO (y)	Julie Simpson via Richard Mann	
D. mel: UAS-myrGFP, QUAS-mtdTomato (3x HA); trans-Tango	Gilad Barnea	
D. mel: UAS(FRT.stop)CsChrimson.mVenus/P{hs-hid}Y; LexAop-FLP/(CyO); ChAT-LexA/TM6b	Matthew Clark	
D. mel: UAS(FRT.stop)CsChrimson.mVenus/P{hs-hid}Y; LexAop-FLP; Gad1-LexA/TM3 sb	Matthew Clark	
D. mel: CLIN: dpn(KDRT.stop)cre.PEST ; Act(loxP.GAL80.stop)lexA::p65, lexAop-rCD2::RFP,UAS-mCD8:: GFP-p10; Stg14KD/TM6B, Tb[+]	Mubarak Syed	RRID:BDSC_67094 and RRID:BDSC_67089
D. mel: norpA^{36};UAS-Chrimson/[CyO.a-GFP];10x UAS-GFP	this study	
D. mel: norpA^{36};tsh-Gal80/cyo-agfp;UAS-Chrimson-mVenus	this study	
D.mel: w^{1118}; s/cyo; UAS-GtACR1-EYFP/TM6b	Claude Desplan	
20xUAS-IVS-phiC31	Tom Clandinin	RRID:BDSC 84155
20xUAS-SPARC2-I-Syn21-CsChrimson::tdTomato	Tom Clandinin	RRID:BDSC 84144
D. mel: norpA^{36} (backcrossed in w118)	Alvarez-Salvado et al., 2018	
