# Supplementary material for: A neural circuit for wind-guided olfactory navigation
Source: Nat Commun. 2022 Aug 8;13:4613. doi: 10.1038/s41467-022-32247-7 (PMC9360402; doi:10.1038/s41467-022-32247-7)
Supplement: Supplementary file 3 — Reporting Summary [file 41467_2022_32247_MOESM3_ESM.pdf]

nature portfolio

Corresponding author(s): Katherine Nagel

Last updated by author(s): May 27, 2022

Reporting Summary

Nature Portfolio wishes to improve the reproducibility of the work that we publish. This form provides structure for consistency and transparency in reporting. For further information on Nature Portfolio policies, see our Editorial Policies and the Editorial Policy Checklist.

Statistics

For all statistical analyses, confirm that the following items are present in the figure legend, table legend, main text, or Methods section.

n/a	Confirmed	
☐	☒	The exact sample size (*n*) for each experimental group/condition, given as a discrete number and unit of measurement
☐	☒	A statement on whether measurements were taken from distinct samples or whether the same sample was measured repeatedly
☐	☒	The statistical test(s) used AND whether they are one- or two-sided<br>*Only common tests should be described solely by name; describe more complex techniques in the Methods section.*
☐	☒	A description of all covariates tested
☐	☒	A description of any assumptions or corrections, such as tests of normality and adjustment for multiple comparisons
☐	☒	A full description of the statistical parameters including central tendency (e.g. means) or other basic estimates (e.g. regression coefficient) AND variation (e.g. standard deviation) or associated estimates of uncertainty (e.g. confidence intervals)
☐	☒	For null hypothesis testing, the test statistic (e.g. *F*, *t*, *r*) with confidence intervals, effect sizes, degrees of freedom and *P* value noted<br>*Give P values as exact values whenever suitable.*
☒	☐	For Bayesian analysis, information on the choice of priors and Markov chain Monte Carlo settings
☒	☐	For hierarchical and complex designs, identification of the appropriate level for tests and full reporting of outcomes
☐	☒	Estimates of effect sizes (e.g. Cohen's *d*, Pearson's *r*), indicating how they were calculated

Our web collection on statistics for biologists contains articles on many of the points above.

Software and code

Policy information about availability of computer code

Data collection	Custom Labview (2014), Matlab (2014-2022), and Python 3.8 code was used to run behavior, electrophysiology, and imaging experiments. Imaging data was collected using ThorImageLS 4.1. Code to run the behavior experiments can be found at https://github.com/nagellab/AlvarezSalvado_ElementaryTransformations.
Data analysis	Custom Matlab code was used to analyze behavioral and imaging data. FIJI used to analyze confocal images. Custom code used to analyze data is available on Github at https://github.com/nagellab/Mathesonetal2022.

For manuscripts utilizing custom algorithms or software that are central to the research but not yet described in published literature, software must be made available to editors and reviewers. We strongly encourage code deposition in a community repository (e.g. GitHub). See the Nature Portfolio guidelines for submitting code & software for further information.

Data

Policy information about availability of data

All manuscripts must include a data availability statement. This statement should provide the following information, where applicable:

- Accession codes, unique identifiers, or web links for publicly available datasets
- A description of any restrictions on data availability
- For clinical datasets or third party data, please ensure that the statement adheres to our policy

All data generated during the study have been deposited at Dryad at doi:10.5061/dryad.qnk98sfj1. The neurprint hemibrain is available at https://neuprint.janelia.org (hemibrain version 1.1)

Human research participants

Policy information about studies involving human research participants and Sex and Gender in Research.

Reporting on sex and gender	n/a
Population characteristics	n/a
Recruitment	n/a
Ethics oversight	n/a

Note that full information on the approval of the study protocol must also be provided in the manuscript.

Field-specific reporting

Please select the one below that is the best fit for your research. If you are not sure, read the appropriate sections before making your selection.

☒ Life sciences ☐ Behavioural & social sciences ☐ Ecological, evolutionary & environmental sciences

For a reference copy of the document with all sections, see nature.com/documents/nr-reporting-summary-flat.pdf

Life sciences study design

All studies must disclose on these points even when the disclosure is negative.

Sample size	Sample sizes for behavioral experiment were based on Alvarez-Salvado et al. 2018. Sample size for imaging and electrophysiology were based on Suver et al. 2019 and Currier et al. 2020.
Data exclusions	For behavior experiments, any trials with tracking errors, or where the fly moved less than 25mm overall were discarded from further analysis. Any fly which moved on less than 5 trials for a condition was excluded as well. For all measured parameters (see below) time periods when the fly was stationary (moving at less than 1mm/s) were omitted. For SPARC experiments, Data points where the fly was walking slower than 1 mm/s or was positioned within 3 mm of the arena walls were excluded from analysis. For imaging experiments, we excluded any flies where we were unable to obtain 5 trials of each direction, either due to fat migration or cell death. We excluded 2 flies from the 65C03-GAL4 data which showed rhythmic spike like activity and did not respond to any phase of our stimulus. For VT062617-GAL4 imaging we excluded 1/17 flies as no columns showed had an average response >2STD above baseline. For electrophysiology we excluded recordings where the access to input ratio was less than 5:1.
Replication	For behavioral experiments, 20-30 flies of each genotype were run. For imaging and electrophysiology, 5-10 flies were imaged or recorded. Where possible, each technique used in the paper was validated by replicating existing findings from the literature (e.g. effects of silencing or activation, response to odor) before extending to new lines.
Randomization	All stimuli for behavior and imaging were presented in random order within a block of trials. Our experimental design did not include sorting of organisms into control and experimental groups. In general all comparisons are based on genotype and all flies of a given genotype were included in each sample.
Blinding	All analyses for behavior and imaging data were automated and applied equally to all genotypes make genotype blinding unnecessary. The same code was applied to all genotypes to produce the analysis, therefore the experimenter's knowledge of the genotype was irrelevant to the outcome of the experiment.

Reporting for specific materials, systems and methods

We require information from authors about some types of materials, experimental systems and methods used in many studies. Here, indicate whether each material, system or method listed is relevant to your study. If you are not sure if a list item applies to your research, read the appropriate section before selecting a response.

Materials & experimental systems

n/a	Involved in the study
☐	☒ Antibodies
☒	☐ Eukaryotic cell lines
☒	☐ Palaeontology and archaeology
☐	☒ Animals and other organisms
☒	☐ Clinical data
☒	☐ Dual use research of concern

Methods

n/a	Involved in the study
☒	☐ ChIP-seq
☒	☐ Flow cytometry
☒	☐ MRI-based neuroimaging

Antibodies

Antibodies used

chicken anti-GFP Fisher Scientific RRID:AB_1074893
mouse anti-nc82 DSHB RRID:AB_2314866
rabbit anti-DsRed Clontech 632496
Rabbit anti-GABA Sigma RRID:AB_477652
Alexa488-conjugated goat anti-chicken Fisher Scientific RRID:AB_2534096
Alexa633-conjugated goat anti-mouse Fisher Scientific RRID:AB_2535719
Alexa568-conjugated goat anti-rabbit Fisher Scientific RRID:AB_2315774
Alexa568-conjugated streptavidin Fisher Scientific RRID:AB_2576217

Validation

Antibodies were validated by staining driver lines with known expression. Antibodies were used only to identify the anatomy of labeled neurons and not to identify molecular species or localization.

Animals and other research organisms

Policy information about studies involving animals; ARRIVE guidelines recommended for reporting animal research, and Sex and Gender in Research

Laboratory animals

Drosophila aged 2-21 days; genotypes as follows:
Figure 1, S1
norpA36/y;UAS-Chrimson-mVenus/orco-GAL4;UAS-10xGFP/+ genetically blind male flies expressing Chrimson under the orco promoter 3-10 days vinegar experiments in Fig. 1C,D,E Fig S1A
norpA36/y;tsh-gal80/orco-GAL4,IR8a-GAL4;UAS-Chrimson-mVenus/+ genetically blind male flies expressing Chrimson under the orco and IR8a promoters 3-10 days Optogenetic activation experiments in Fig. 1C,D,E Fig. S1C
norpA36/y;UAS-Chrimson-mVenus;UAS-10xGFP x X-GAL4 or split-GAL4

X-GAL4: orco-GAL4,IR8a-GAL4 (II), empty-GAL4 (II), empty split-GAL4 (II,III), OR59a (III), OR42a (III), IR75a (III), IR64a (II), OR92A (III), OR42b (II) genetically blind hemizygous male flies expressing Chrimson under various GAL4 or split-GAL4 drivers on chromosomes II or III. 3-10 days optogenetic activation experiments in Fig. 1E Fig. S1A,B,D
norpA36/y;+/UAS-Chrimson-mVenus;+/UAS-10xGFP parental control 3-10 days parental control for activation in Fig. 1E
UAS-TNT5905 parental control; UAS-TNT backcrossed 5 generations to w1118 5905 3-10 days parental control for silencing in Fig. 1F
UAS-TNT5905 x X-GAL4

X-GAL4: orco-GAL4, IR8a-GAL4, orco-GAL4,IR8a-GAL4 flies with various ORNs silenced 3-10 days Fig. 1F,G, Fig. S1E
Figure 2, S2
norpA36/y;tsh-Gal80;UAS-Chrimson-mVenus x X-GAL4 or split-GAL4

X-GAL4/split-GAL4: LH1538, LH1396, LH1539, MB082C, MB077B, MB052B, MB434B, MB112C, MB011B, MB543B, MB050B, MB018B, MB027B, MB549C, LH2193, LH989, LH290. genetically blind hemizygous male flies expressing Chrimson under various GAL4 or split-GAL4 drivers on chromosomes II or III. tsh-Gal80 included to suppress VNC expression. 3-10 days Fig. 2A-D, Fig. S2A,B
UAS-TNT5905 x X-GAL4 or split-GAL4

X-GAL4: LH1538, LH1396, LH1539, MB077B, MB052B, MB112C or + for parental control flies with MB/LH neurons silenced 3-10 days Fig. S1C
+;UAS-GCaMP6f;UAS-tdTomato x X-GAL4

X-GAL4: LH1396, MB052B, MB077B, MB082C, ss47432 (LNa) GCaMP6f and tdTOM expressed in LHAd1b2 using the LH1396 split GAL4 driver or in MBONs15-19 using MB052B, MBON12 using MB077B, or MBON 13,14 using MB082C, LNa using ss47432 5-21 days Fig. 2E, Fig. S2E
+;MB027B-GAL4AD/+;UAS-10xGFP/MB027B-GAL4DB expression of GFP in ’3 MBONs for electrophysiology 1-3 days Fig. 2F
Figure 3, S3
UAS-myrGFP, QUAS-mtdTomato (3x HA); trans-Tango x X-GAL4

X-GAL4: LH1396, MB052B Trans-tango driven by LH1396 and MB052B 10-20 days; raised at 19°C Fig. 3A
norpA36/y;tsh-Gal80;UAS-Chrimson-mVenus x X-GAL4 or split-GAL4

Dorsal Inputs: VT056792-GAL4, VT002458-GAL4, 84C10-GAL4, VT027955-GAL4, 58F01-GAL4, 58F02-GAL4, 71A02-GAL4,VT026663-GAL4, VT20633-GAL4, VT004849-GAL4, 23E10-GAL4, 26B11-GAL4, 28H10-GAL4, 12D12-GAL4, 65C03-GAL4, 45D04-GAL4, LH2392

Ventral Inputs: VT036875-GAL4, 33E06-GAL4, VT056509-GAL4, 72A04-GAL4, 78G09-GAL4, vt049652-GAL4, 13B10-GAL4, VT033929-GAL4, VT046276-GAL4, VT041421-GAL4,VT029515-GAL4

PFN: 44B10-GAL4; VT039497-GAL4,16D01-GAL4, 67B06-GAL4, SS52577, SS52244, SS54549, SS02255, SS00239, 43D09AD; VT000986DB

Split-GAL4: 43D09-AD;65C03-DB, 12D12-AD;VT027955-DB, VT026663AD;65C03DB, 12D12AD;65C03DB, VT041421AD;28H10DB, VT041421AD;33E06DB, VT041421DB;65H10DB, VT029515AD; 78G09DB, LH1478, 13B10AD;VT029515DB, 13B10AD; VT041421DB

Blank: Empty-GAL4, Empty Split-Gal4 genetically blind hemizygous male flies expressing Chrimson under various GAL4 or split-GAL4 drivers on chromosomes II or III. tsh-Gal80 included to suppress VNC expression. 3-10 days Fig. 3B, C, S3
norpA36/y;tsh-Gal80;UAS-Chrimson-mVenus x X-GAL4 or split-GAL4

Additional CX neurons: 19C06-GAL4, 65H10-GAL4, VT024599-GAL4, VT058487-GAL4, VT000986-GAL4, 24E05-GAL4, VT062617-GAL4, 43D09-GAL4, VT019352-GAL4, VT030322-GAL4, 94G02-GAL4, VT050238-GAL4, VT063191-GAL4, VT060736-GAL4, 43D09AD;VT062617DB, VT063948-GAL4,
46G06-GAL4, V000624-GAL4, VT060202-GAL4, 73A06-GAL4,
VT037489-GAL4, VT020739-GAL4, VT032906-GAL4, 60D05-GAL4, SS50464, SS02718, SS50420
Figure 4, S4
CLIN x UAS-Chrimson-mVenus;21D07-GAL4 21D07>Chrimson-mVenus filtered through CLIN to restrict expression to type II (CX) neurons 3-10 days Fig. 4A (anatomy),C (behaviour) Fig. S4A
+;UAS-GCaMP6f;UAS-tdTomato x X-GAL4

X-GAL4: 21D07-GAL4, 65C03-GAL4, vFB split (13B10AD;VT041421DB), 12D12-GAL4 GCaMP6f and tdTOM expressed under the various FB drivers 5-21 days Fig. 4B,D imaging, Fig. S4D,E
+;UAS-GCaMP6f;UAS-tdTomato x X-GAL4

X-GAL4: MB052B, MB077B, MB082C, LH1396, 21D07-GAL4, 65C03-GAL4, vFB split (13B10AD;VT041421DB), 12D12-GAL4 GCaMP6f and tdTOM expressed under the various drivers 5-21 days Fig. 4E
norpA36/y;tsh-Gal80;UAS-Chrimson-mVenus x X-GAL4 or split-GAL4

X-GAL4: 65C03-GAL4, 12D12-GAL4, vFB split (13B10AD;VT041421DB), VT029515-GAL4 genetically blind hemizygous male flies expressing Chrimson under various GAL4 or split-GAL4 drivers on chromosomes II or III. tsh-Gal80 included to suppress VNC expression. Fig. 4A (anatomy),C (behavior), Fig. S4A
UAS(FRT.stop)-Chrimson-mVenus; LexAop-FLP; ChAT-LexA; ChAT-LexA x X-GAL4 or split-GAL4

X-GAL4: 21D07-GAL4, 65C03-GAL4, vFB split (13B10AD;VT041421DB), 12D12-GAL4 cross to identify cholinergic neurons within a GAL4 or split-GAL4 line 3-20 days Fig. S4B
UAS(FRT.stop)-Chrimson-mVenus; LexAop-FLP; Gad1-LexA x X-GAL4 or split-GAL4

X-GAL4: 65C03-GAL4, 12D12-GAL4 cross to identify GABAergic neurons within a GAL4 or split-GAL4 line 3-20 days Fig. S4B
UAS-TNT5905 x X-GAL4 or split-GAL4

X-GAL4: 65C03-GAL4, 45D04-GAL4, VT029515-GAL4, vFB split (13B10AD;VT041421DB) flies with FB neurons constitutively silenced 3-10 days Fig. S4C
Figure 5, S5
+;UAS-GCaMP6f;UAS-tdTomato x VT062617-GAL4 GCaMP6f and tdTOM expressed in hΔC neurons 5-21 days Fig. 5D-H, S5A
UAS(FRT.stop)-Chrimson-mVenus; LexAop-FLP; ChAT-LexA; ChAT-LexA x VT062617-GAL4 cross to identify cholinergic neurons within a GAL4 line 3-20 days Fig. S5A
UAS(FRT.stop)-Chrimson-mVenus; LexAop-FLP; Gad1-LexA x VT062617-GAL4 cross to identify GABAergic neurons within a GAL4 line 3-20 days Fig. S5A
Figure 6, S6
20XUAS-SPARC2-I-Syn21-CsChrimson::tdTomato x

UAS-phiC31;VT062617-GAL4
UAS-phiC31;empty-GAL4 15% expression of Chrimson using SPARC2-I in hΔC neurons or empty-GAL4 3-7 days Fig. 6 A-E, S6A-B
norpA36/y;tsh-Gal80;UAS-Chrimson-mVenus x X-GAL4 or split-GAL4

X-GAL4: VT062617-GAL4, hΔC split1 (19G02AD;VT062617DB), hΔC split2 (VT024634AD;
VT062617DB) genetically blind hemizygous male flies expressing Chrimson under various GAL4 or split-GAL4 drivers on chromosomes II or III. tsh-Gal80 included to suppress VNC expression. 3-10 days Fig. 6D-F
norpa36/+;s/+;X-GAL4/UAS-GTACR1
or
norpa36/+;s/X-GAL4; UAS-GTACR1/+

X-GAL4(II): orco-GAL4, IR8a-GAL4
X-GAL4(III): 21D07-GAL4, VT062617-GAL4
Acute silencing of ORNs, FB5AB, and hΔC with GtACR, in genetically blind hemizygous males 3-10 days Fig. 6G-H

Wild animals

No wild animals were used in this study.

Reporting on sex	For optogenetic activation experiments, experiments were run in male norpA hemizygotes, which are genetically blind, to eliminate any possible innate visual responses to red light. All other flies used were female. We detected no difference in olfactory behavior of male versus female flies in our assay in a previous study (Alvarez-Salvado et al., 2018).
Field-collected samples	No field collected samples were used in this study.
Ethics oversight	Only invertebrate organisms were used in this study, therefore no animal protocol was required.

Note that full information on the approval of the study protocol must also be provided in the manuscript.
